# Expression of HER2 in the eye and the potential for on-target side effects with antibody-drug conjugates

**DOI:** 10.1038/s41598-026-51353-w

**Published:** 2026-05-07

**Authors:** Simon Davenport, Bin Xie, Kevin Hamblett, Diana Hausman, Ivonne Villalobos, Hardeep P. Singh, David Cobrinik, David S. B. Hoon, Jian-Yuan Zhou, Maria Sibug-Saber, Yanling Ma, Gottfried E. Konecny, Martin Heur, Dennis J. Slamon, Michael F. Press

**Affiliations:** 1 Department of Pathology, Norris Comprehensive Cancer Center, University of Southern California, Los Angeles, CA USA; 2 Zymeworks Inc, Seattle, WA USA; 3 Department of Ophthalmology, Roski Eye Institute, and Norris Comprehensive Cancer Center, The Vision Center and Saban Research Institute, University of Southern California, Children’s Hospital of Los Angeles, University of Southern California, Los Angeles, CA USA; 4 Saint John’s Cancer Institute, Saint John’s Health Center PHS, Santa Monica, CA USA; 5 Department of Medicine, David Geffen School of Medicine at UCLA, Los Angeles, CA USA; 6 Department of Ophthalmology, Keck Medicine of USC, University of Southern California, Los Angeles, CA USA; 7 Ellison Institute of Technology, Los Angeles, CA USA; 8 Present Address: Pfizer, Inc., Bothell, WA USA; 9 Present Address: Link Immunotherapeutics, Inc., Seattle, WA USA; 10 Present Address: Epic Bio, South San Francisco, CA USA

**Keywords:** Cancer, Diseases, Drug discovery, Medical research

## Abstract

The use of antibody-drug conjugates (ADCs) specific to human epidermal growth factor receptor 2 (HER2), such as ado-trastuzumab emtansine (T-DM1), trastuzumab deruxtecan (Enhertu, T-DXd), and trastuzumab duocarmazine (SYD985), to treat breast cancer patients has been associated with a variety of toxicities including corneal epithelial keratitis. The anatomic basis for ocular toxicities has not been established. Using immunohistochemistry, HER2 expression was identified in the human corneal, limbal and conjunctival epithelium of fetal and adult human eyes. We also identified HER2 expression in tissues from the *Macaca fascicularis* (monkey) to confirm a similar distribution of HER2 expression as found in humans and to support the use of this species as a toxicology model in preclinical testing of anti-HER2 ADCs. As in the human, expression of HER2 in monkey was limited to normal epithelium, as illustrated for the eye, kidney, and uterus. Incubation of normal human corneal epithelial cell lines with trastuzumab or T-DXd demonstrated the capacity for receptor-mediated endocytosis of HER2-targeted therapies by epithelia in the eye. Our findings of HER2 expression in normal human corneal epithelium suggest that the ocular adverse events associated with HER2-targeted ADCs may be driven, at least in part, by an on-target effect.

**Supplementary Information:**

The online version contains supplementary material available at 10.1038/s41598-026-51353-w.

## Introduction

HER2 has become one of the most important therapeutic targets in breast cancer. The *ERBB2*/*HER2* gene was reported to be amplified with pathologic overexpression in approximately 20% of human breast cancers with those patients whose cancers had this alteration demonstrating a significantly worse disease-free and overall survival compared to those patients whose breast cancers did not have this alteration^1–4^. Subsequently, women with *HER2*-amplified/overexpressed metastatic breast cancer treated with humanized anti-HER2 antibody (trastuzumab) in combination with chemotherapy demonstrated significantly improved progression-free and overall survival compared to women with *HER2*-amplified/overexpressed metastatic breast cancer treated with chemotherapy alone^5,6^. Similarly, women with *HER2*-amplified/overexpressed early invasive breast carcinoma treated with humanized anti-HER2 antibody (trastuzumab) in combination with chemotherapy demonstrated a significantly improved disease-free and overall survival compared to women treated with the same chemotherapy alone^7–9^. Although some investigators hypothesized that low expression of HER2 protein in breast cancer may serve as an effective therapeutic target for trastuzumab plus chemotherapy, a large clinical trial of women whose breast cancers had low expression of HER2 by immunohistochemistry (IHC 1 + or IHC 2+/FISH-not-amplified) demonstrated no significant change in clinical outcomes for women treated with trastuzumab plus chemotherapy compared to chemotherapy alone^10^.

The addition of antibody-drug conjugates (ADCs), particularly trastuzumab-deruxtecan (T-DXd), to the treatment options for breast cancer patients has provided improved efficacy in patients with HER2-positive breast cancers^11^. Preliminary findings from early phase clinical trials also show therapeutic responses in some patients with HER2-low breast carcinomas^12^. These promising advancements have not been without drawbacks. Alarmingly, several clinical studies have shown various degrees of corneal epithelial side effects are common characteristics of anti-HER2 treatment using antibody-drug conjugates, such as ado-trastuzumab emtansine (T-DM1)^13^, trastuzumab duocarmazine^14^, and trastuzumab-deruxtecan.

Antibody-drug conjugates involve molecular binding of a humanized monoclonal antibody used for targeting a particular cancer-related protein and a cytotoxic agent through a cleavable or non-cleavable chemical linker. After the ADC binds a target cell mediated by its monoclonal antibody moiety, the drug conjugate is internalized via antigen-mediated endocytosis, followed by lysosomal degradation of the ADC and release of the cytotoxic agent to act intracellularly on the cell expressing the target. Several studies have correlated the level of HER2 expression and efficacious response to HER2-targeting ADCs and have offered variable mechanisms for the observed efficacy beyond the primary, “on-target” mechanism described, above^15,16^. Particularly when HER2 expression is low, ADC activity is influenced by “off-target” mechanisms involving extracellular cathepsins, membrane permeability of the payload, antibody-dependent cell cytotoxicity, and antibody-dependent cell phagocytosis with induction of a durable T cell memory when immunogenic cell death is caused by Fcγ-receptor engagement of HER2-ADCs^15,16^.

A previous study from our laboratory investigated expression of the human epidermal growth factor receptor 2 (HER2) in normal human tissues^17^. We demonstrated *HER2* mRNA and HER2 protein throughout normal adult and fetal epithelial cells in all tissues examined. HER2 protein was expressed at low levels on the basal and lateral cell membranes of all examined glandular epithelial cells throughout the body^17^. HER2 protein was also identified on stratified squamous epithelial cells of the skin and cervix. However, human eyes were not included in this original study, with only the retina characterized from fetal eyes.

While current speculation is that the optical side effects of ADCs are non-specific, or “off-target”, due to the release of the ADC drug payload in the circulation or via diffusion from targeted neighboring cells, we hypothesize that the ophthalmic side effects are due, at least in part, to “on-target, off-tumor” effects of the anti-HER2-ADC delivering a toxic agent to an exquisitely sensitive area, the eye. Here, we characterize the specificity and sensitivity of antibodies which we use to localize HER2 in human and animal eyes with immunohistochemical assays and demonstrate the specific internalization of HER2-targeted therapies by normal corneal epithelial cells. These studies demonstrate the distribution of HER2 in the human and monkey eye providing support for the perspective that the anti-HER2 component of the ADC delivers the drug to the cornea creating the potential for toxic side effects.

## Materials and methods

### Human cell lines

Human cancer cell lines SK-BR-3 (RRID: CVCL_0033), JIMT-1 (RRID: CVCL_2077), ZR-75-1 (RRID: CVCL_0588), Capan-1 (RRID: CVCL_0237), and MCF-7 (RRID: CVCL_0031) with known levels of HER2 protein product as well as the RPE-1 (RRID: CVCL_4388) (human retinal pigment epithelium) immortalized cell line were used to confirm the specificity and sensitivity of our HER2 antibodies by western immunoblot (WB) analysis. These cell lines were also used to optimize and validate our immunohistochemical assay methods and were selected due to their known *HER2* gene status and known HER2 expression status^18^. The cell lines were each grown in culture according to conditions previously described^18^. All cell lines were authenticated by analyses of short tandem repeats (STRs) and checked for microorganism contamination prior to the investigation. In order to characterize immunohistochemical (IHC) staining levels using frozen and formalin-fixed, paraffin-embedded (FFPE) cells, the cell lines were collected and processed as either pellets frozen in liquid nitrogen or FFPE samples using standard pathology tissue processing, as summarized elsewhere^3,19^.

HCE-2 (ATCC CRL-3582, RRID: CVCL_3316) and 2.040 pRSV-T (ATCC CRL-3603, RRID: CVCL_6346) normal epithelial cells isolated from the cornea were used to demonstrate the capacity for therapeutic antibody (trastuzumab) and ADC (T-DXd) internalization by non-malignant cells of the human cornea. These cells were grown according to the instructions provided for Keratinocyte Serum-Free Medium (SFM) (Gibco/ThermoFisher #17005-042) in culture vessels pre-coated with bovine serum albumin, bovine collagen type I, and fibronectin.

### Study tissues

Tissue samples, in triplicate, procured for evaluation of HER2 expression included normal cynomolgus macaque eye, normal cynomolgus kidney, normal cynomolgus uterus, and normal cynomolgus brain, as well as normal rabbit eye, normal rabbit kidney, normal rabbit prostate, and normal rabbit brain and were purchased from Covance Laboratories Inc./Labcorp. The fetal human eyes (*n* = 3), gestational weeks 16–18, were obtained from Advanced Bioscience Resources, Inc. (Alameda, CA) (IRB CCI-13–00048), and deidentified adult eyes were purchased from BioIVT LLC (*n* = 3) or remnant specimens donated for corneal transplantation (*n* = 5). Limited clinicopathologic history was available for the procured tissues. However, no indications of pre-existing ocular maladies were provided; the cause of death was unrelated to the eye.

Although the eyes of human subjects were involved in this study and were approved by our Institutional Review Board (IRB HS-20–00795), none of these study subjects were included in any clinical trial and, therefore, Consolidated Standards of Reporting Trials (CONSORT) do not apply to this investigation. The de-identified subjects were more than 18 years of age. The National Institutes of Health (NIH) and the Office for Human Research Protections (OHRP) do not consider research involving only coded private information or specimens to involve human subjects as defined under 45 CFR 46.102(f) if the following conditions are both met: the private information or specimens were not collected specifically for the currently proposed research project through an interaction or intervention with living individuals and the key to decipher the code is destroyed before the research begins. Our study met these criteria.

### Western immunoblot analyses

Western immunoblot analyses were performed using A0485 and 10H8 anti-HER2 antibodies, as described elsewhere in detail^2,20^. The A0485 primary antibody is a component of the HercepTest kit (Dako #SK001, RRID: AB_2935822). The 10H8 anti-HER2 antibody is a mouse monoclonal antibody developed in our laboratory^20^ that we routinely use as part of a laboratory-developed IHC assay for HER2 assessments^21–23^.

In brief, protein lysates were prepared using equal numbers of cells (100 µL/10^6^ cells) in RIPA buffer (Sigma #R0278) and protease inhibitors (Sigma #P8340). Wells in 7.5% polyacrylamide gels were loaded with equal amounts of protein lysates (15 µL) in loading buffer and subjected to SDS-polyacrylamide gel electrophoresis at a constant 200 V for 48 min. Molecular weight Chameleon Duo protein markers (Licor #928–60000) were included with the samples. Transfer to nitrocellulose membranes was performed at 100 V for 60 min in tris-glycine buffer containing 20% methanol. Membranes were incubated with primary anti-HER2 antibody for 1 h at room temperature, diluted in TBS- or PBS-based blocking buffer (Licor #927–80001 or #927–90001) containing 10% Tween-20. Membranes were washed and treated with secondary antibody (Licor #925–32211 or #925–32210) for one hour at room temperature followed by four washes with tris-buffered saline Tween (TBST). Each immunoblot was scanned with identical acquisition settings using LI-COR ODYSSEY CLx Imager with Image Studio Software Ver 5.2.

A semi-quantitative analysis of HER2 expression in each cancer cell line was performed using Empiria Studio Software developed by LI-COR. The target signal fluorescence intensity of each cancer cell line band at approximately 185 kilodaltons (kDa) was normalized using REVERT Total Protein Stain (Licor #926–11010). The HER2 expression level, or “fold change”, of each cell line was calculated by dividing each normalized signal intensity by the normalized signal intensity of the immortalized retinal pigment epithelial (RPE-1) cell line as the control.

### HER2 protein expression by IHC

The A0485 and 10H8 anti-HER2 antibodies were used in our immunohistochemical studies. The commercially available A0485 primary antibody was originally generated by immunizing rabbits with synthetic HER2 protein (Dako, Inc). The 10H8 monoclonal antibody was the product of mice immunized first with HER2 extracellular domain peptides and, subsequently, with live mouse (3T3) cells engineered to overexpress human HER2 protein^20^.

Immunohistochemical localization of HER2 was performed in frozen tissue sections and FFPE tissue sections according to methods previously described^20^, except that a secondary anti-rabbit antibody conjugated to horseradish peroxidase (HRP) (Dako #P044801-2, RRID: AB_2617138) or anti-mouse antibody with HRP linked by a dextran-polymer (Dako #K4001, RRID: AB_2827819) was used to replace peroxidase-antiperoxidase secondary and tertiary antibodies^21–23^. Normal immunoglobulin reagent controls, or antibody diluent in place of primary antibody, were used to confirm the immunostaining specificity.

IHC using the 10H8 anti-HER2 monoclonal antibody was performed in FFPE tissues and cell lines without antigen retrieval as described elsewhere^21–23^. In brief, after quenching endogenous peroxidase activity with hydrogen peroxide (3% H_2_O_2_ in PBS) the procedure involves the sequential application of the 10H8 monoclonal antibody (10 µg/mL, 60 min, room temperature) to tissue sections followed by a goat anti-mouse immunoglobulin conjugated to an HRP–labeled dextran polymer (30 min, room temperature).

IHC using the A0485 anti-HER2 polyclonal antibody (6 µg/mL, 60 min, room temperature) was performed similarly. However, heat-induced epitope retrieval was performed using a solution of sodium citrate and surfactant, buffered to pH 6 (component of Dako #SK001), at 99 °C for 40 min. The secondary antibody used to identify rabbit immunoglobulins was purchased from Dako (Dako #P044801-2) and was incubated for 30 min at room temperature at a concentration of 2.5 µg/mL.

Immunohistochemistry with 10H8 in frozen tissue sections was performed similarly to immunohistochemistry in FFPE tissues and cell line pellets without modification.

The site of immunoprecipitates was identified using a chromogen, diaminobenzidine, visualized microscopically. Immunostaining was scored subjectively with an Olympus bright-field microscope as 0, 1+, 2+, and 3 + as described^21–23^. Photomicrographs of stained sections were captured using the same Olympus brightfield microscope and a camera, or with a Zeiss AxioImager M2 and Axiocam 807 color camera using Zeiss Zen software Ver 3.8.

### Epitope mapping of 10H8 antibody

10H8 is an IgG2a monoclonal mouse antibody that recognizes an extracellular domain of the human epidermal growth factor receptor 2 (HER2) protein^20^. Enzyme-linked immunosorbent assay (ELISA), using short, synthesized peptides, was employed to identify the specific epitope of the HER2 protein recognized by the 10H8 antibody. Peptides spanning the extracellular domains of HER2 were custom ordered from Invitrogen:

SFDGDPAS

SFDGDPA

  FDGDPASN

   DGDPASN

      GDPASNT

Competitive ELISA was performed to determine the strongest 10H8:peptide pair.

The amino acid sequence of the homologous rabbit HER2 protein varies by 2 positions within the 10H8 epitope. This variation proved sufficient to prevent 10H8 antibody interaction with normal rabbit tissues.

Earlier work cloned 19 *HER2* PCR fragments of varying length spanning from nucleotide position 1 through 1793 of the *HER2* ECD into a His-tagged expression vector and transformed *E. coli* was used to generate peptides to screen by western blotting for 10H8 binding via the GST-tag (membranes were probed with anti-GST antibodies). Once the suspected epitope was reduced to < 15 amino acids, competitive ELISA using synthesized peptides and purified 10H8 antibody was performed.

### Internalization assays

Prior to conducting the internalization assays, HER2 expression was demonstrated in HCE-2 and 2.040 pRSV-T normal corneal cells using a variation of the IHC described above using the anti-HER2 10H8 primary antibody and goat anti-mouse AF568 secondary antibody (ThermoFisher #A-11001). SK-BR-3 cells were included as a positive control for the assay. Cells were cultured and immunostained in 6-well plates on glass coverslips. The coverslips were carefully inverted and applied to glass slides for imaging on a Zeiss AxioPlan2 microscope with a Zeiss MRm AxioCam black and white camera and Zeiss AxioVision software for multidimensional acquisition.

HCE-2 and 2.040 pRSV-T were plated on coverslips in 6-well plates with complete SFM and allowed to adhere for 24 h. A 4X working solution of Trastuzumab (Biosimilar, CST #10583), T-DXd (MedChemExpress #HY-138298 A), or Human IgG Isotype Control (Invitrogen #31154) was prepared at 24 µg/mL (160nM) in SFM and combined with equal volume of 4X Zenon pHrodo Red Human IgG Labeling Reagent (ThermoFisher #Z25612) and incubated for 5 min to prepare a 2X staining solution. 1 mL of the 2X staining solution was applied to each well prior to adding 1 mL of 2X membrane impermeant NucSpot 470 Nuclear Stain (Biotium #40083) diluted in SFM. The coverslips were carefully inverted and applied to glass slides for imaging on a Zeiss AxioPlan2 microscope with a Zeiss MRm AxioCam black and white camera and Zeiss AxioVision software for multidimensional acquisition with autoexposure at multiple timepoints.

### Institutional Review Board

An application (HS-20–00795) was approved by the University of Southern California’s Institutional Review Board for the entire scope of the project. The methods utilized and experiments performed while conducting this study were done so according to the relevant guidelines and regulations and as described in the approved IRB application. Fetal eyes were obtained under Children’s Hospital Los Angeles Institutional Review Board protocol CCI-13–00048. No live humans or animals were involved in this study. Informed consent from individuals providing tissues was provided to the collecting site, including those purchased from a commercial vendor, BioIVT.

## Results

### Validation of anti-HER2 antibodies using western blot and IHC in cell lines with known HER2 gene amplification/expression status

**Fig. 1:**
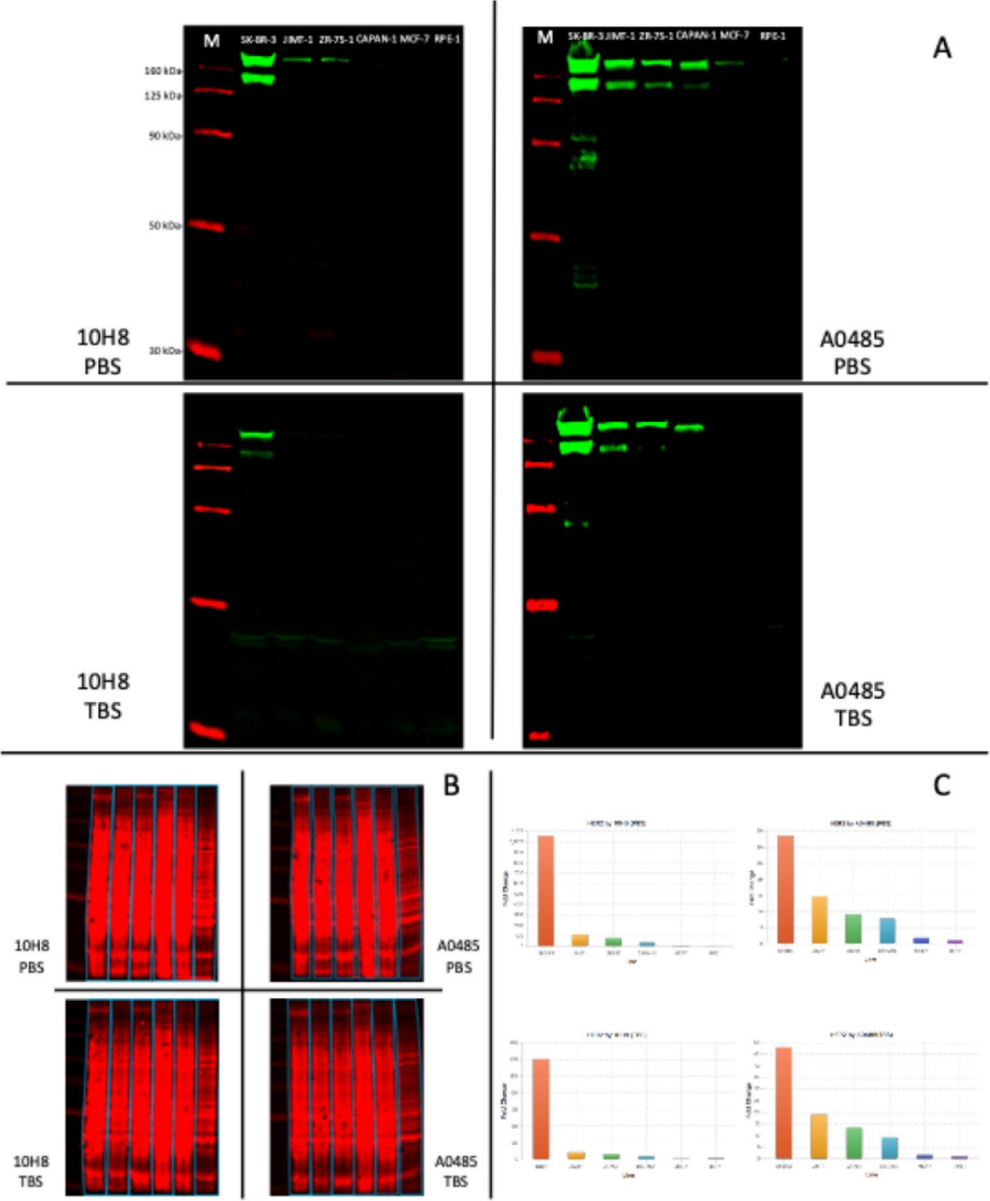
Western Immunoblot Analyses Using 10H8 and A0485 Anti-Human HER2 Antibodies in Human Carcinoma Cell Lines. **(A)** Western blot analyses of 10H8 and A0485 anti-HER2 antibodies demonstrated specificity for HER2 protein bands of approximately 185 kDa and 150 kDa for both antibodies under varying PBS and TBS buffer conditions. **(B)** Total protein stain demonstrates equivalent loading of protein lysates across the lanes. **(C)** Target signal normalization in B facilitates quantitation of p185 protein bands in each WB lane demonstrating relative quantities of HER2 protein in each cell line. The western blot lanes have been loaded as follows: lane M, molecular weight markers; lane 1, SK-BR-3 human breast cancer cells; lane 2, JIMT-1 human breast cancer cells; lane 3, ZR-75-1 human breast cancer cells; lane 4, CAPAN-1, human pancreas adenocarcinoma cells; lane 5, MCF-7 human breast cancer cells; lane 6, RPE-1 human retinal pigment epithelial cells. Supplemental file 1 is included with overexposed representations of each membrane to illustrate the full distribution of protein electrophoresis and that no cropping or obscuration of bands was performed. The largest proteins represented on the membranes exceed the 260 kDa band included in the molecular weight ladder.

**Fig. 2:**
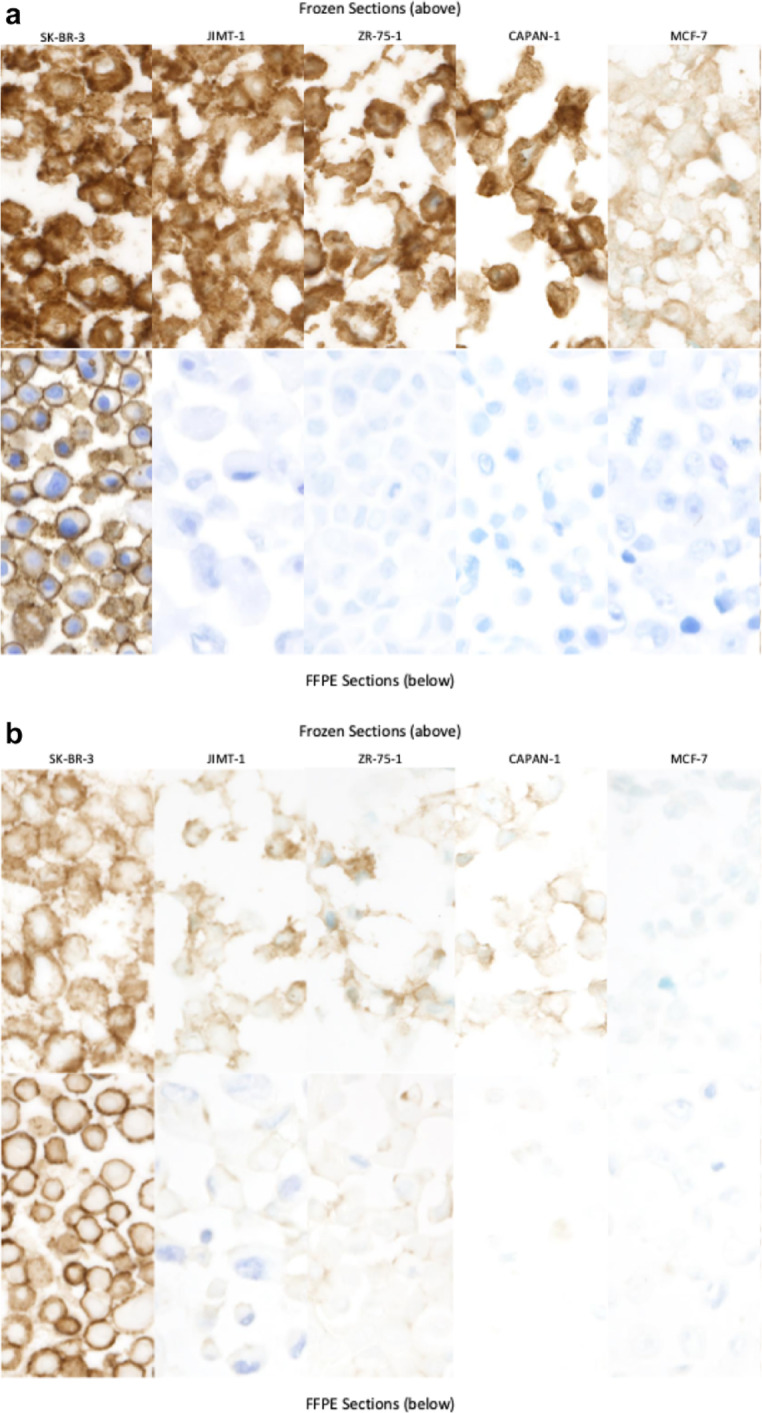
Immunohistochemical localization of HER2 protein in frozen, compared to formalin-fixed, paraffin-embedded, human breast cancer cell lines (SK-BR-3, JIMT-1, ZR-75-1, and MCF-7) as well as a human pancreatic carcinoma (CAPAN-1) cell line. **(A)** Localization of HER2 in frozen human cancer cells (above) or the same cell lines fixed in formalin and paraffin-embedded (below) using the 10H8 anti-human HER2 antibody. **(B)** Localization of HER2 in frozen human cancer cells (above) or the same cell lines fixed in formalin and paraffin-embedded (below) using the A0485 anti-human HER2 antibody for all cell lines (all images obtained with 40X objective). Normal IgG controls (not illustrated) demonstrated no detectable immunohistochemical staining.

Human carcinoma cell lines (SK-BR-3, JIMT-1, ZR-75-1, Capan-1, and MCF-7) were grown in sufficient quantities to prepare lysates for western blots, frozen cell line samples for immunohistochemistry and formalin-fixed, paraffin-embedded (FFPE) cells for routine immunohistochemistry evaluations. Western immunoblot analyses confirmed that both 10H8 and A0485 antibodies specifically recognized a protein band of approximately 185 kDa, consistent with HER2 protein recognition, as well as a second band of approximately 150 kDa previously considered to be an alternatively spliced HER2 protein product^20^(Fig. 1A). Quantitation of the p185 protein band demonstrated high levels of expression only in the SK-BR-3 human breast cancer cell line, which is known to have *HER2* gene amplification and overexpression^3,17,18^. Similarly, immunohistochemical localization of the HER2 protein product performed with 10H8 and A0485 antibodies demonstrated that 10H8 functioned effectively in this assay to demonstrate membrane immunostaining using either frozen cell lines or FFPE cell lines, particularly without antigen retrieval (Table 1; Fig. 2A).

**Table 1:** Subjective scoring (IHC 0, 1+, 2+, 3+) of HER2 immunohistochemistry.

Cell Line	A0485 Frozen	A0485 FFPE	10H8 Frozen	10H8 FFPE
**SK-BR-3**	2+/3+	3+	3+	3+
**JIMT-1**	1+/2+	1+/2+	2+/3+	0
**ZR-75-1**	1+/2+	1+/2+	2+	0
**CAPAN-1**	1+	1+	2+	0
**MCF-7**	0/1+	0/1+	2+	0

Immunohistochemical staining was performed using either A0485 or 10H8 as the primary anti-HER2 antibody in the IHC procedure with or without antigen retrieval as described in Materials and Methods (see Fig. 2). The diaminobenzidine reaction product was evaluated by brightfield microscopy and scored subjectively according to intensity of membrane staining varying from no detectable staining (0), weak (1+), intermediate (2+), strong (3+) immunostaining, or a range of staining intensities (2+/3+, etc.).

In contrast, A0485 required antigen retrieval (heat-induced antigen retrieval at pH 6) to localize HER2 in FFPE cell lines that had HER2 expression and did not successfully localize HER2 protein in frozen cell pellets or in FFPE cell lines without the use of antigen retrieval (Fig. 2B).

### Expression of HER2 protein by immunohistochemistry in human and animal tissues

Tissue specimens were evaluated for HER2 membrane protein expression by immunohistochemistry with 10H8 anti-HER2 antibody in frozen tissue sections. The immunohistochemistry analyses demonstrated HER2 expression successfully in the human and cynomolgus monkey tissues, but not in the rabbit tissues. Because the rabbit tissues were not successfully immunostained with 10H8 antibody, we presumed the HER2 extracellular domain recognized by 10H8 in humans may not be conserved in rabbits (*Oryctolagus cuniculus*). To address this possibility, we mapped the epitope recognized by 10H8 using ELISA with multiple amino acid peptides. We found that the 10H8 antibody recognized a HER2 extracellular epitope in humans (SFDGDPASN) that is not conserved (SF**E**GDPAS**A**) in rabbits (*Oryctolagus cuniculus*).

Regarding the A0485 rabbit monoclonal antibody, we considered the requirement of utilizing an anti-rabbit IgG HRP-conjugated secondary antibody in the IHC procedure to localize the primary A0485 antibody, a technical contraindication that prevented reliable discrimination of HER2 protein in tissue samples from endogenous rabbit immunoglobulins with this antibody. This is due to the potential for the secondary anti-rabbit antibody used in the IHC procedure to recognize not only A0485 primary anti-HER2 antibody, but also rabbit immunoglobulins present in the tissue sample. Finally, the intensity of immunostaining by antibody A0485 in frozen cell lines and tissues was so weak that we considered it to be insufficient for a convincing demonstration of expression of HER2 protein. Therefore, immunostaining of rabbit tissues with this primary antibody was not completed.

**Fig. 3:**
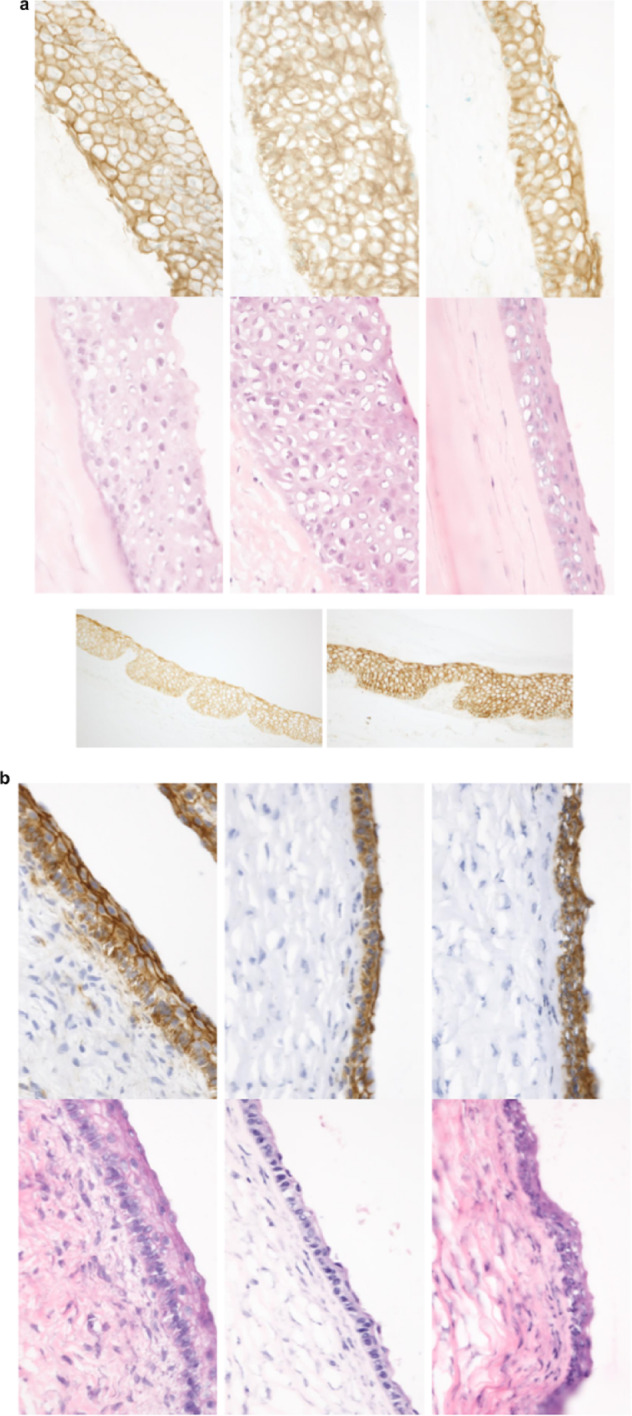
Immunohistochemical localization of HER2 protein in the cornea of adult and fetal human eyes. **(A)** Expression of HER2 protein, identified by Immunohistochemistry in the cell membranes of squamous epithelial cells overlying the cornea from three adult human eyes. Localization of HER2 protein with 10H8 in three separate frozen human corneas (above) demonstrates expression of HER2 throughout the full thickness of the epithelium. Hematoxylin and eosin-stained sections of the same frozen eyes (below) demonstrate the normal histologic appearance of the cornea (*N* = 3). Lower power photomicrographs at the bottom of the figure of tangential cross-sections of the limbus provide the reader with an ocular landmark, the papillary architecture of the palisades of Vogt. **(B)** HER2 protein is localized by immunohistochemistry to cell membranes of in the squamous epithelium of the cornea in three fetal human eyes (above). Localization of HER2 protein with 10H8 in three separate frozen human corneas (above) demonstrates expression of HER2 throughout the full thickness of the epithelium. The histology of the same eyes stained with hematoxylin-and-eosin is illustrated below (all images obtained with 40X objective). Normal IgG negative controls (not illustrated) demonstrated no detectable immunohistochemical staining. The palisades of Vogt architecture was not identified in the fetal eye samples; it is likely the structures will not have yet fully formed until postnatal development.

**Fig. 4:**
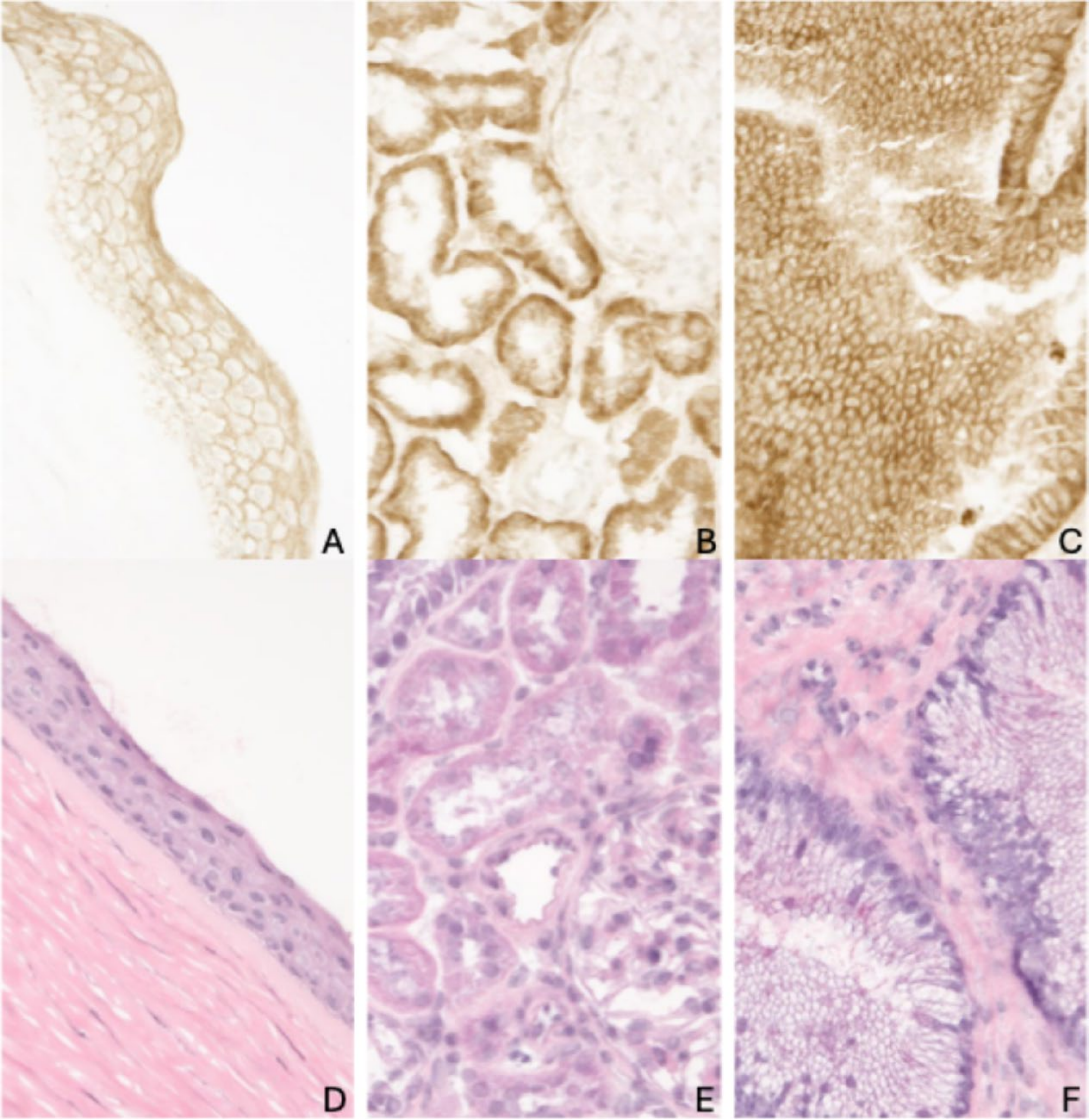
Immunohistochemical localization of HER2 protein in select normal cynomolgus monkey (*Macaca fascicularis*) tissues. **(A)** Squamous epithelium of the cornea from a normal eye demonstrates HER2 localization with 10H8 by IHC. **(B)** Similarly, HER2 is localized to tubule epithelial cells, but not glomeruli, of the kidney. **(C)** Endometrial epithelial cells of the uterus also demonstrate membrane localization of HER2 by immunohistochemistry with 10H8 antibody. **D – F.)** Hematoxylin-and-eosin-stained tissue sections demonstrate normal histological appearance. Normal IgG negative controls (not illustrated) demonstrated no detectable immunohistochemical staining.

Using optimized conditions for HER2 IHC in frozen tissues with the 10H8 anti-HER2 antibody, we identified HER2 expression in the human corneal, limbal and conjunctival epithelium of the human eye in adult eyes (Fig. 3A) and fetal eyes (Fig. 3B). We also analyzed selected normal tissues from the *Macaca fascicularis* (monkey) to confirm a similar distribution of HER2 expression as found in humans, and to support the use of this species as a toxicology model in preclinical testing of anti-HER2 ADCs. As in the human, expression was limited to normal epithelium, as demonstrated in the eye, kidney, and uterus (Fig. 4). The subjective interpretation of HER2 immunostaining intensity is remarkably uniform among and within each sample. This pattern of uniformity among HER-expressing cells is readily appreciated when immunostaining frozen tissues, but contrasted by immunostaining patterns observed in FFPE samples where immunostaining intensity and frequency is negatively impacted by prolonged formaldehyde fixation, dehydration, and paraffin embedding.

### Internalization assays

**Fig. 5:**
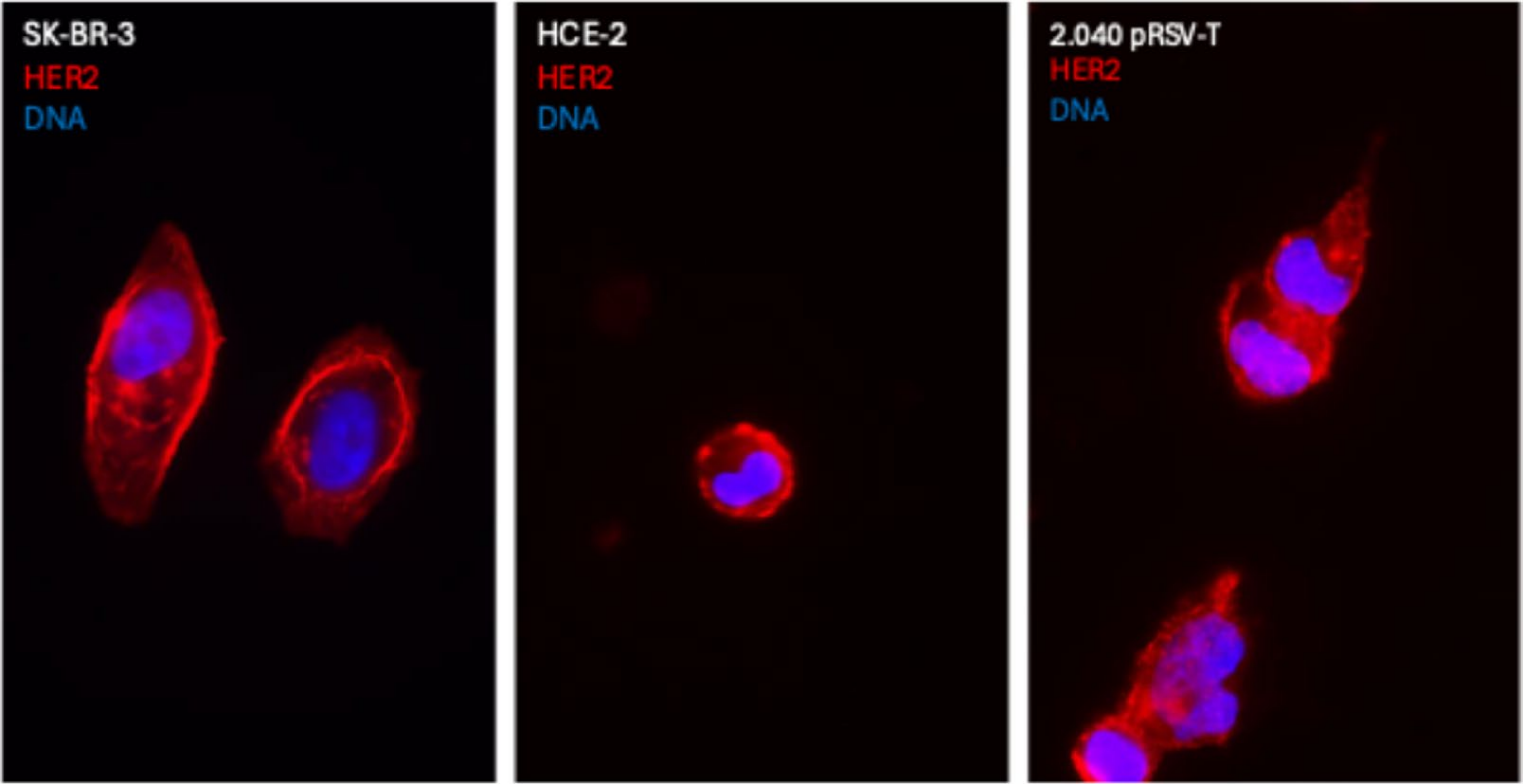
Immunofluorescent localization of HER2 protein using 10H8 anti-HER2 primary antibody and AF568 goat anti-mouse secondary antibody on whole cells grown on coverslips in 6-well plates. The fluorescence intensity emitted by SK-BR-3 cells was significantly greater than both HCE-2 and 2.040 pRSV-T cells. The difference in intensity is obscured by the AxioVision software automatic exposure setting for each fluorescence channel (the camera exposure time was significantly shorter while acquiring SK-BR-3 stained cells versus HCE-2 or 2.040 pRSV-T cells). The NucSpot fluorescence of DNA is pseudo-colored blue.

**Fig. 6:**
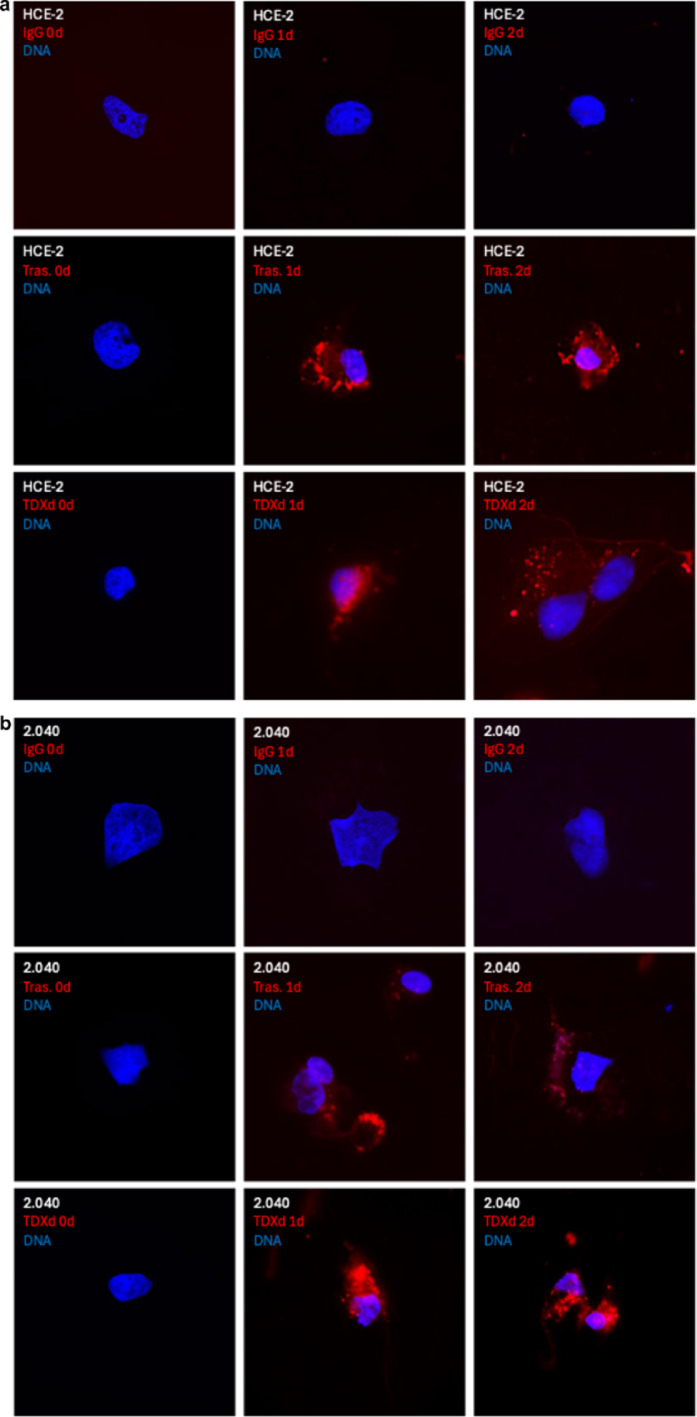
Fluorescence of labeled trastuzumab and T-DXd after incubation with normal corneal cells demonstrating receptor-mediated endocytosis of both treatments. No fluorescence was observed upon incubation (0d) or with normal human IgG isotype control, but significant fluorescence was observed after 24 h (1d) and 48 h (2d), indicating endosomal/lysosomal ingestion of trastuzumab and T-DXd. Trastuzumab and T-DXd are both labeled in red. The NucSpot fluorescence of DNA is pseudo-colored blue. **(A)** HCE-2 normal corneal cell line. **(B)** 2.040 pRSV-T normal corneal cell line.

Immunofluorescent staining of HCE-2 and 2.040 pRSV-T using the 10H8 anti-HER2 primary antibody confirmed HER2 expression by these normal cells at an expectedly lower level of expression than the SK-BR-3 cell line harboring *HER2* gene amplification (Fig. 5). Despite the low levels of HER2 expression by these normal corneal cell lines, both demonstrated the ability to perform receptor-mediated endocytosis of trastuzumab and T-DXd (Fig. 6A and B). Negligible pinocytic uptake of a normal human IgG isotype control was observed.

The labeling reagent, Zenon pHrodo Red, uses a pHrodo Red-labeled Fab fragment directed against the Fc portion of an intact IgG primary antibody to form a labeling complex. The fluorescence of the labeling dye dramatically increases as the pH of the local environment decreases (endosomal pH ~ 6.3–5.5, lysosomal pH ~ 4.7). Thus, antibody coupling to the pHrodo Red dye provides for observation of endocytosis of labeled antibodies via microscopy or flow cytometry, distinct from antibody-receptor binding. The NucSpot membrane-impermeant fluorescent DNA stain labeled all nuclei with compromised membrane integrity. No fluorescence by labeled trastuzumab or T-DXd was detected upon incubation (0d, within 2 h) prior to endocytic uptake. The fluorescence detected in each cell line increased over time and photomicrographs were captured after 1 day (22–26 h) and 2 days (46–50 h) of cells incubated with labeled trastuzumab or T-DXd. Fluorescence did not increase when the cell lines were incubated with the labeled human IgG isotype control demonstrating receptor-specific uptake, not by non-specific FcR-mediated uptake.

## Discussion

In the current study, we confirmed the specificity of both A0485 and 10H8 for the p185 kDa HER2 protein using both western immunoblot analyses and immunohistochemistry in human carcinoma cell lines with known HER2 expression levels in both formalin-fixed and fresh frozen cell pellets. In addition, for the 10H8 antibody, specificity for the extracellular domain of human HER2 was confirmed by epitope mapping to demonstrate that human and monkey, but not rabbit, HER2 was recognized because of amino acid sequence differences in the epitope between human (SFDGDPASN) and rabbit (SF**E**GDPAS**A**) HER2 at the 10H8 binding site.

Our studies demonstrate expression of HER2 protein in the cell membrane of human and monkey corneal epithelial cells, and that trastuzumab and T-DXd not only bind immortalized human corneal epithelial cells but are taken up by these benign cells through receptor-mediated endocytosis, i.e. “on-target, off-tumor” binding. The specific internalization of each therapeutic served as the endpoint of the experiment. Cell cytotoxicity was not quantified or demonstrated, albeit cells treated with T-DXd were clearly impacted by topoisomerase I inhibition as strands of DNA could be microscopically observed protruding from affected nuclei.

Although ocular surface adverse events are relatively common in clinical trials of the antibody-drug conjugates T-DM1, SYD985, and T-DXd, there is a relative lack of published evidence regarding ocular toxicity in preclinical models. Therefore, we examined the eyes of primates used in these models to assess HER2 expression as well as select normal tissues to determine if the distribution of expression appeared to be similar to the distribution in human tissues. HER2 was identified by IHC in frozen macaque tissues and the distribution was similar to that of HER2 in humans, suggesting that primates may experience similar visual disturbances that are not currently monitored.

Ocular adverse events have been commonly associated with the administration of antibody-drug conjugates (ADCs) in general. Of the twelve currently FDA-approved ADCs, three have “black box” warnings for ocular toxicity including mirvetuximab soravtansine (Elahere), tisotumab vedotin (Tivdak), and the recently withdrawn blenantamab mafadotin (Blenrep). Mirvetuximab soravtansine was approved for treatment of folate receptor alpha (FOLR1)-positive, platinum-resistant epithelial ovarian carcinomas. Tisotumab vedotin targets tissue factor (TF-011) expressing tumors and blenantamab mafadotin is designed to treat B-cell maturation antigen (BCMA) expressing multiple myeloma. However, to our knowledge expression of none of these therapeutic targets has been characterized in the eye. The ocular adverse events associated with mirvutuximab soravtansine have been largely hypothesized to be due to target-independent effects of the DM4 payload, as other ADCs containing the DM4 payload also report ocular adverse events^24^.

Various degrees of corneal epithelial side effects are also common characteristics of anti-HER2 antibody-drug conjugates (Table 2). Ocular adverse events have been reported for ado-trastuzumab emtansine (T-DM1)^13^, trastuzumab-deruxtecan (T-DXd)^25^ as well as for trastuzumab duocarmazine^26^, ARX788^27^, and A166^28^, all of which use the same HER2 targeting antibody, trastuzumab, but differ in their payload, linker and/or conjugation chemistry (Table 2). The efficacy and toxicity profiles of each are greatly influenced by target expression, membrane permeability of the ADC payload (potential “bystander effect”), linker chemistry and the expression of enzymes, like cathepsins, with the capacity to cleave the linkers within the tumor microenvironment^15,16^. In contrast to the anti-HER2 ADC examples noted above, there have been no reported ocular adverse events reported for RC48^29^.

**Table 2:** Ocular Side effects associated with anti-HER2 antibody-drug conjugate therapies.

ADC Drug	Ocular Side Effects	Antibody/Target/Disease	Linker	Payload	Reference
Trastuzumab-emtansine (T-DM1)	Not Reported	Trastuzumab/HER2 + Breast cancer	N-maleimidomethyl- cyclohexane-1-carboxylate (mcc) (thioether, stable)	Maytansinoid emtansine (DM1)	Krop IE et al., *J Clin Oncol*. 2010.
T-DM1	31%	Trastuzumab/HER2 + Breast cancer	thioether (stable)	DM1	Burris et al., *J Clin Oncol*, 2011
T-DM1	46%	Trastuzumab/HER2	thioether (stable)	DM1	Beeram et al., *Cancer*, 2012
T-DM1	Case Report (corneal lesions)	Trastuzumab/HER2 + Breast cancer	N-maleimidomethyl- cyclohexane-1-carboxylate (stable)	DM1	Tsuda et al., *J Cornea*, 2016.
T-DM1	100%* (30% symptomatic)	Trastuzumab/HER2	thioether (stable)	DM1	Deklerck et al., BCRT, 2019
Trastuzumab-duocarmazine (SYD985)	71%* (104 of 146 patients)	Trastuzumab/HER2	cleavable linker	Duocarmycin–hydroxybenzamide–azaindole	Banjeri et al., *Lancet Oncol*, 2019
Disitamab vedotin (RC48-ADC)	Not Reported	Hertuzumab/HER2 + Adv Cancers	valine–citrulline (vc) linker (cleavable)	MMAE	Xu Y et al., *Gastric Ca*, 2021
PF-06804103	9.7% keratitis	Trastuzumab/HER2	(cleavable)	Aur0101 (Auristatin)	Meric-Bernstam F et al., *Mol Cancer Ther*, 2023
Trastuzumab-deruxtecan (T-DXd)	Dry eye (11%)	Trastuzumab/HER2 + breast cancer	(cleavable)	Topoisomerase I inhibitor (DXd)	Modi S et al. NEJM, 2020, DESTINY Breast01
Trastuzumab-deruxtecan (T-DXd)	Not Reported	Trastuzumab/HER2 + breast cancer	(cleavable)	DXd	Cortes J et al. NEJM, 2022, DESTINY Breast03
Trastuzumab-deruxtecan (T-DXd)	Not Reported	Trastuzumab/HER2 + breast cancer	(cleavable)	DXd	Modi S et al. NEJM, 2022, DESTINY Breast04
ARX788	corneal epitheliopathy (46.4%)	Trastuzumab/HER2 + breast cancer	synthetic amino acids para-acetylphenylalanine (pAF)	AS269	Zhang J et al., *Clin Cancer Res*, 2022
A166	corneal epitheliopathy (84%), blurred vision (74%)	Trastuzumab amino acid sequence/HER2 breast and non-breast	Stable protease-cleavable valine citrulline linker.	Duostatin-5	Zhang J et al., npj *Breast Cancer*, 2023

(HER2) human epidermal growth factor receptor 2; (T-DM1) trastuzumab emtansine; (T-DXd) trastuzumab deruxtecan; (RC-48) disitamab vedotin.

*Ophthalmological examinations performed on all patients.

The most commonly reported ocular adverse events in patients treated with T-DM1, SYD985, or T-DXd involving the ocular surface included dry eye, blurred vision, visual impairment, punctate keratitis, and conjunctivitis^14,24,26,30^. The frequency of ocular adverse events reported in clinical trials varies from 10% to 100%^14,26,28,30^ (Table 2). Although a majority of these adverse events are mild (grade 1 or grade 2), high-grade (grade 3 or grade 4) events have been reported^14,28^. Our findings of HER2 expression in normal human ocular epithelium are consistent with an on-target effect for trastuzumab-directed therapy impacting normal tissues that express low, but detectable levels of HER2 protein. Clinical outcomes following ocular adverse events are not consistently reported, nor are they consistently identified^13^. However, most ocular side effects improved or resolved with discontinuation of therapy or with topical steroid treatment.

Corneal findings have been reported in numerous patients who have been treated with anti-HER2 antibody-drug conjugates (Table 2). Recent studies have shown corneal epithelial changes are characteristic of anti-HER2 treatment using ADCs, such as ado-trastuzumab emtansine^13^(T-DM1), trastuzumab duocarmazine^14^, and trastuzumab deruxtecan^11,12^(T-DXd) (Table 2). However, other HER2 ADCs including disitamab vedotin (a.k.a. RC48-ADC)^29^ and PF-06804103 did not report high levels of ocular toxicity^31^ with only ~ 10% of patients having keratitis. Although the degree of variance across HER2 ADC studies is noteworthy, the frequency of ocular toxicity is highest in clinical studies that have systematically performed ophthalmological examinations in all patients on study^13,14^, while others have reported patient symptomatic complaints without performing ocular exams of all patients^11,12,31,32^. Many of the patients treated with anti-HER2 ADC experience asymptomatic ocular effects that are apparent on ophthalmological examination^13^. Corneal findings include changes characterized as “corneal epithelial microcysts” when observed by in ophthalmological slit lamp examination^13^.

While the mechanism of ADC toxicity is not well-documented, some investigators have hypothesized that ADC instability and premature payload release into the circulation may be the primary attribute responsible for the maximal tolerated dose and dose-limiting toxicity reported for ADCs^31^. However, the levels of circulating free payload are documented to be consistently low with either a non-cleavable linker as in T-DM1 (free plasma DM1 consistently < 10 ng/mL, minimum quantifiable concentration of 0.737 ng/mL for DM1)^26^ or a cleavable linker as in the PF-06804103 anti-HER2-ADC (free PF-06380101, also consistently < 10 ng/mL)^31^. By molar equivalents, systemic exposure of DM1 was more than 50-fold less than that of T-DM1^26^ and exposure to free PF-06380101 payload was more than 16,000-fold less than PF-06804103 anti-HER2-ADC^31,33^. Interestingly, the authors of the study reporting one of the highest levels of ocular toxicity attempted to address this issue as follows: “since the Cmax of the A166 ADC at 6.0 mg/kg was approximately the same level as that of T-DXd at 5.4 mg/kg and T-DM1 at 3.6 mg/kg, the Cmax of the free payload was approximately 9% and 13% of that of T-DXd and T-DM1, respectively, indicating that A166 may have lower off-target toxicity under the same ADC exposure.”^28^ How these low levels of circulating free payload would preferentially be distributed to the relatively avascular cornea is not clear. In contrast, the intact anti-HER2-ADC does have an on-target, off-tumor substrate with which to bind in the HER2-expressing cornea, as demonstrated by this study.

Our study assessed expression of HER2 protein in the normal human eye, particularly the cornea, compared to expression levels in preclinical toxicology models to determine if HER2 expression could be identified in the eye and, therefore, might serve as an on-target source for HER2-targeted ADCs that have been associated with ocular adverse events. We hope this study serves to clarify the often-misunderstood expression profile of HER2 by demonstrating it is expressed throughout normal epithelia, and those cells retain the capacity for HER2-ADC internalization. Additionally, this demonstration impacts common misconceptions in the field: that “HER2-negative” is a misnomer, as homozygous deletion of the *HER2* gene has not been identified and is likely embryonic-lethal; and “HER2-heterogeneity” is an extremely rare phenomenon^34^. These contentious and important conclusions are due to technical shortcomings when evaluating FFPE samples rather than an optimized assay using frozen samples as demonstrated in this study and are critical for meaningful understanding of the mechanisms by which these therapeutics act, their limitations, their toxicity profiles, and the standard and prophylactic care intended patients require.

## Electronic Supplementary Material

Below is the link to the electronic supplementary material.

Supplementary Material 1

## Data Availability

No datasets were generated during and/or analyzed during the current study. Detailed methods, certain reagents, and raw image files are available from the corresponding author on reasonable request.
