# Supplementary figures and images for: Expression of HER2 in the eye and the potential for on-target side effects with antibody-drug conjugates
Source: Sci Rep. 2026 May 7;16:21731. doi: 10.1038/s41598-026-51353-w (PMC13358114; doi:10.1038/s41598-026-51353-w)

A

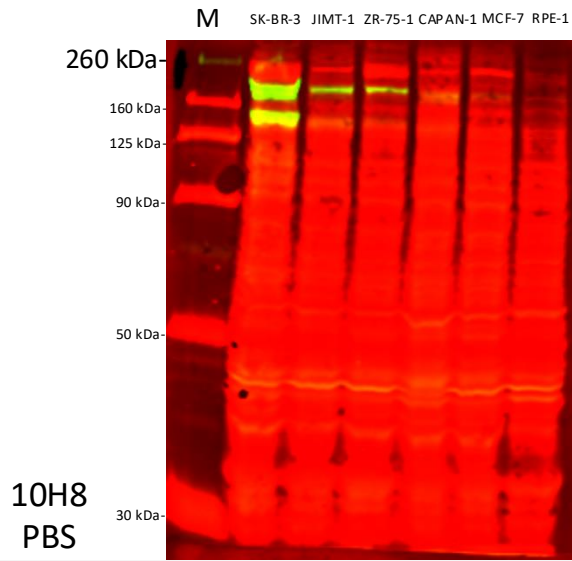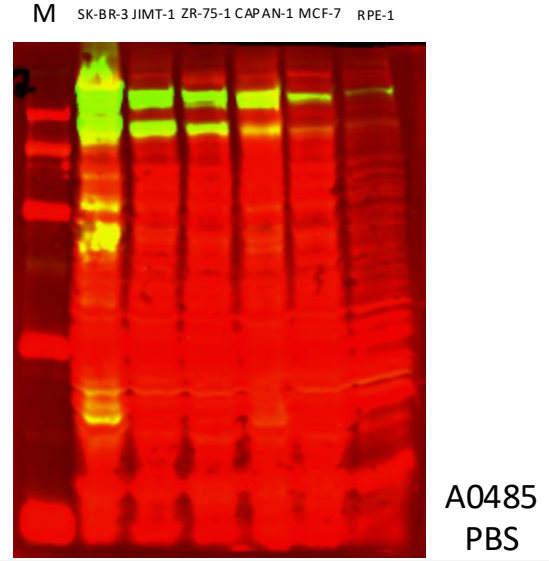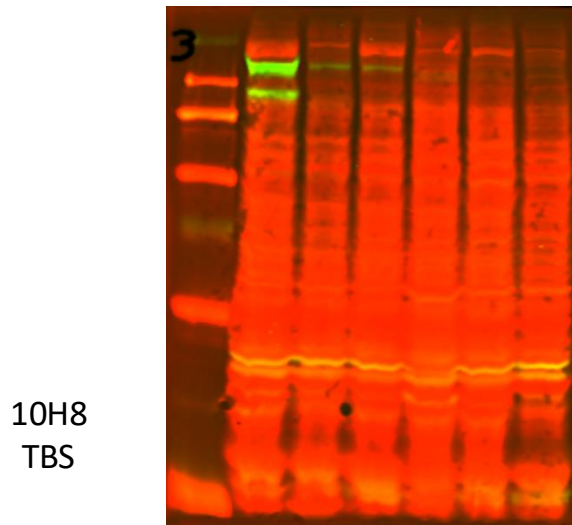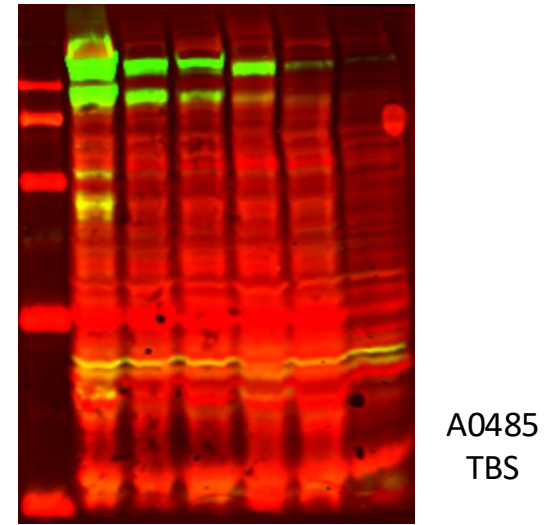

Supplement: Supplementary file 1 — Supplementary Material 1 [file 41598_2026_51353_MOESM1_ESM.pdf]
